# Intravascular Imaging versus Physiological Assessment versus Biomechanics—Which Is a Better Guide for Coronary Revascularization

**DOI:** 10.3390/diagnostics13122117

**Published:** 2023-06-19

**Authors:** Miłosz Starczyński, Stanisław Dudek, Piotr Baruś, Emilia Niedzieska, Mateusz Wawrzeńczyk, Dorota Ochijewicz, Adam Piasecki, Karolina Gumiężna, Krzysztof Milewski, Marcin Grabowski, Janusz Kochman, Mariusz Tomaniak

**Affiliations:** 1 First Department of Cardiology, Medical University of Warsaw, Banacha 1a Str., 02-097 Warsaw, Poland; starczynskimilosz@gmail.com (M.S.); stanislaw.dudek0@wp.pl (S.D.); piotrbarus@op.pl (P.B.); emilia.niedzieska@gmail.com (E.N.); mateusz.wawrzenczyk@gmail.com (M.W.); dorota.ochijewicz@gmail.com (D.O.); adam1piasecki@gmail.com (A.P.); kgumiezna@gmail.com (K.G.); marcin.grabowski@wum.edu.pl (M.G.); janusz.kochman@wum.edu.pl (J.K.); 2 Center for Cardiovascular Research and Development, American Heart of Poland, 43-316 Bielsko-Biała, Poland; krzysztof.milewski@ahop.pl

**Keywords:** fractional flow reserve, coronary artery disease, percutaneous coronary intervention, optical coherence tomography, intravascular ultrasound, instantaneous wave-free ratio, quantitative flow ratio, computed tomography-derived flow fractional reserve, angiography-derived fractional flow reserve, optical flow ratio

## Abstract

Today, coronary artery disease (CAD) continues to be a prominent cause of death worldwide. A reliable assessment of coronary stenosis represents a prerequisite for the appropriate management of CAD. Nevertheless, there are still major challenges pertaining to some limitations of current imaging and functional diagnostic modalities. The present review summarizes the current data on invasive functional and intracoronary imaging assessment using optical coherence tomography (OCT), and intravascular ultrasound (IVUS). Amongst the functional parameters—on top of fractional flow reserve (FFR) and instantaneous wave-free ratio (iFR)—we point to novel angiography-based measures such as quantitative flow ratio (QFR), vessel fractional flow reserve (vFFR), angiography-derived fractional flow reserve (FFRangio), and computed tomography-derived flow fractional reserve (FFR-CT), as well as hybrid approaches focusing on optical flow ratio (OFR), computational fluid dynamics and attempts to quantify the forces exaggerated by blood on the coronary plaque and vessel wall.

## 1. Introduction

Coronary artery disease (CAD) continues to be a prominent cause of mortality worldwide. The primary diagnosis of this disease includes coronary artery imaging, to initiate relevant therapeutic management decisions, which mainly focus on revascularization. Coronary artery visualization techniques can be divided into two groups—invasive and non-invasive. Revascularization using percutaneous coronary intervention (PCI) or coronary artery bypass grafting (CABG) restores epicardial blood flow and, consequently, relieves symptoms of angina and related myocardial ischemia [1].

Apart from angiography, a well-established gold standard in the field of morphological imaging, among modern coronary invasive assessing techniques we distinguish primarily fractional flow reserve (FFR), optical coherence tomography (OCT) and intravascular ultrasound (IVUS). The mechanisms behind them differ and concentrate on the diverse issues regarding CAD pathophysiology which provides a characterization of different properties of lesions to provide efficacious personalized treatments.

**Figure 1:**
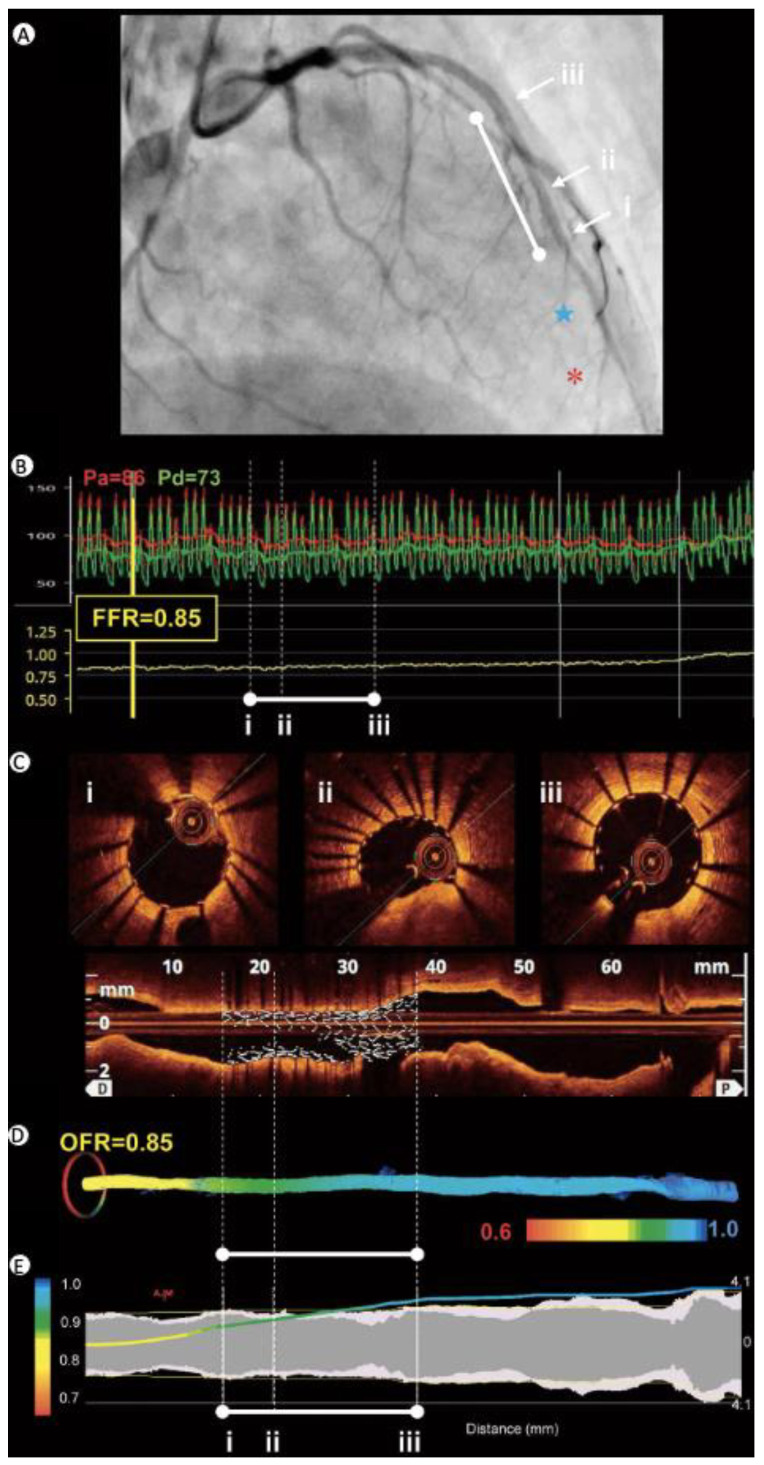
Different techniques used in the assessment of coronary stenosis after PCI: (**A**) coronary angiogram, white line = stent (diameter 2.5 mm and length 23 mm) in mid LAD, red asterisk = FFR measurement site, blue star = OFR measurement site; (**B**) FFR = 0.85, in-stent ∆FFR = 0.05; (**C**) OCT at distal reference, MSA (2.78 mm^2^) site, and proximal reference; (**D**) color-coded OFR on the 3D-OCT lumen view. OFR = 0.85, in-stent ∆OFR = 0.06; (**E**) OFR, pullback curve and lumen diameters (short diameter in grey and long diameter in white). 3D, three dimensional; FFR, fractional flow reserve; LAD, left anterior descending artery; MSA, minimum stent area; OCT, optical coherence tomography; OFR, optical flow ratio; PCI, percutaneous coronary intervention. Reproduced under a Creative Commons license from [6].

Angiography presents itself as a two-dimensional image, which is a limiting issue considering the complex three-dimensional structure of coronary vessels [2]. Another restriction is that angiography acts as a luminogram, showing only changes in the lumen, without the possibility to visualize coronary artery wall morphology [3]. What is more, this technique does not assess the lesion severity regarding physiological significance. It seems to be particularly problematic in cases of intermediate stenoses, which are defined as a 40–70% vessel obstruction, where angiography detects less than half of them [4]. This inability to distinguish which lesions may lead to ischemia within the myocardium and an urge to perform revascularization has encouraged the adoption of other coronary assessing techniques (Figure 1) [5,6].

In this article, we provide brief characteristics of the different techniques used in the assessment of coronary stenosis which indicates therapeutic management. Our article contains a review of invasive and non-invasive techniques for the visualization of coronary stenosis, to evaluate the diagnostic values of procedures accompanying PCI.

## 2. Fractional Flow Reserve

The FFR is an invasive method of assessment of coronary blood flow disturbances in a stenotic artery. It is defined as an index of maximal blood flow in a stenosed coronary vessel to the theoretical normal maximal flow, i.e., in the absence of the obstructive lesion, in the same distribution. This ratio is calculated by measuring distal coronary pressure behind an obstruction and dividing it by either proximal coronary pressure or aortic pressure [7]. A coronary flow must be determined during maximal blood flow (hyperemia) with a measured pressure ratio. Only under this condition coronary vessel flow is stable, vascular resistance is reduced to a minimum and a linear relationship between blood flow and pressure can be obtained [8,9]. In a non-stenotic vessel, the FFR expected score is 1.0. According to numerous studies, values above 0.75 allow for safely deferring PCI implementation. However, many recent studies support using 0.80 rather than 0.75 as a threshold below which percutaneous intervention is indicated [10]. In the current guidelines of myocardial revascularization of the European Society of Cardiology (ESC), FFR equal to 0.80 (iFR equal to 0.89) is described as the standardized cut-off value in the estimation of hemodynamic relevance of coronary stenosis [11]. The ESC recommends FFR assessment for coronary artery lesions with intermediate stenosis of 40–90% to provide an efficacious treatment strategy for affected vessels in patients without evidence of ischemia in non-invasive testing or in those with multivessel disease [12,13].

Steady-state of maximal hyperemia can be induced through administering multiple agents in which adenosine plays a pivotal role, but nitrates are also reported in the literature. Before adenosine was introduced into general use, papaverine had been the agent of choice in the assessment of coronary blood flow. Its use has been replaced with adenosine because of concerns about complications, such as QT prolongation [14]. Hyperemic agents can be administered either intravenously or intracoronary (IC) as a bolus. Peripheral and central vein infusion is associated with a more frequent occurrence of significant hemodynamic changes and transient symptoms, such as chest discomfort, chest pain, and shortness of breath, in comparison to intracoronary adenosine [15]. On the other hand, the intracoronary route of administration can provoke atrioventricular blocks, but it is rarely observed in clinical practice [14]. 

**Figure 2:**
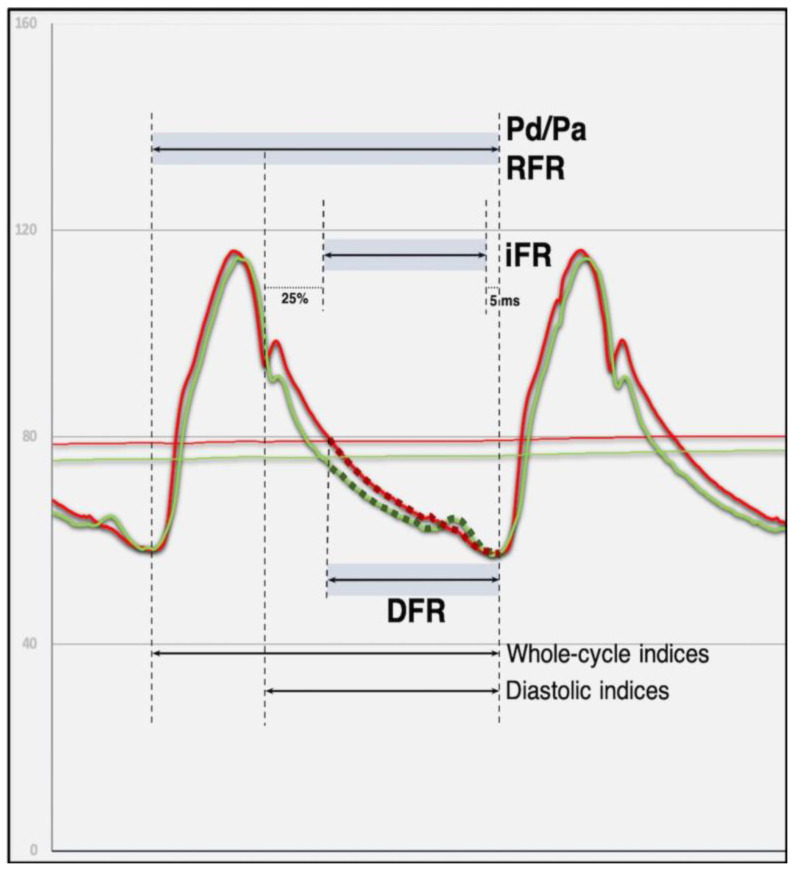
Schematic representation of the commonly available non-hyperemic pressure ratios and the periods of the cardiac cycle from which they are calculated. Pd/Pa, distal coronary pressure to aortic pressure ratio; iFR, instantaneous wave-free ratio; RFR, resting full-cycle ratio; DFR, Diastolic Hyperaemia-Free Ratio. Reproduced under a Creative Commons license from [19].

Taking into consideration the adverse effects of hyperemia-inducing agents, investigators tried to define a variant of FFR that would be independent of the maximal vasodilatation procedure. Sen et al. in their Adenosine Vasodilator Independent Stenosis Evaluation (ADVICE) study compared flow and pressure measurements during a cardiac cycle, when adenosine was infused with a cardiac cycle, and when the hyperemic agent was not used. The purpose of such a designed trial was to find a certain period during the cycle, when vascular resistance is naturally minimalized and stable, despite the absence of adenosine. This interval was defined during diastole and a new pressure-derived index of stenosis severity, that does not require pharmacologic vasodilation, was termed instantaneous wave-free ratio (iFR) [16]. iFR was the primary method of measuring pressure without hyperemia. When asked if there was another measure as useful for the determination of the pressure ratio, Van’t Veer et al. showed that each interval gave numerically almost identical results to iFR [17]. Newer non-hyperemic pressure ratio (NHPR) methodologies, such as the full resting cycle ratio (RFR), the diastolic hyperemia-free ratio (DFR), and the diastolic pressure ratio (dPR) have been found to be consistent with the iFR and thus can be used interconvertible for clinical use. The dPR is defined as the mean Pd/Pa during the entire diastole. The DFR is defined as the mean Pd/Pa while Pa is less than the mean Pa with a negative slope. The RFR is defined as the lowest average Pd/Pa during the entire heart cycle [18]. The analysis corroborated that there were no relevant differences in the values of pressure ratios at rest during the entire cycle (Pd/Pa and RFR) and diastolic (dPR and mathematically derived iFR) (Figure 2 and Table 1) [19,20]. Whole-cycle NHPRs show better repeatability and clinical precision after PCI than diastolic NHPRs, possibly due to less disturbance caused mainly by diastolic reactive hyperemia and left ventricular stunning. Studies have confirmed that NHPRs have similar diagnostic efficacy to FFR before PCI to forecast long-term outcomes [20]. A series of studies were conducted to evaluate the correlation between conventional FFR and iFR. They proved that iFR is not inferior to FFR regarding the primary endpoint, defined as the rate of major cardiovascular adverse events for 1 year of clinical outcomes [21,22]. Moreover, among the group with FFR guidance, a significantly higher rate of procedural adverse signs and symptoms was reported [21]. 

## 3. Non-Invasive Fractional Flow Reserve Indices

Despite the indisputable value of the FFR assessment, which has made it the gold standard for the physiological evaluation of intermediate stenoses, this modality was not a widely adopted technique. Several limitations, such as generating additional expenses through the cost of the pressure wire and the obligatory adenosine administration, contributed to this occurrence [23]. This issue is well-reflected by a 2014 survey research, that in clinical practice, a large percentage of clinicians base the decision solely on angiography, despite the possibility of using other modalities, indicating a troubling lack of consistency between clinical practice and guidelines [24]. This prompted the researchers to approach FFR in a different way.

**Figure 3:**
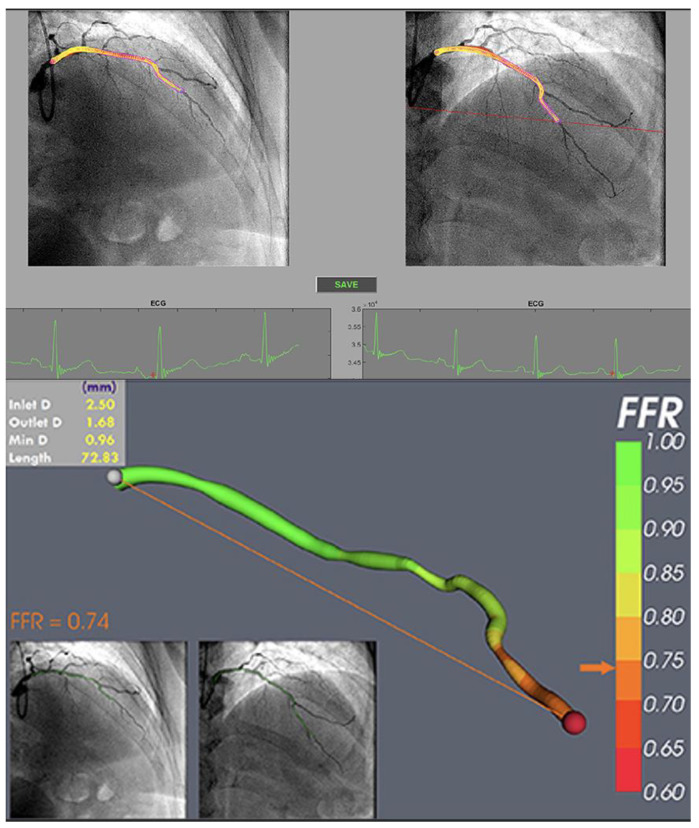
vFFR result after 3D reconstruction CFD simulation showing a vFFR in the LAD of 0.74. vFFR, vessel fractional flow reserve; LAD, left anterior descending artery. Reproduced and adapted under a Creative Commons license from [33].

Quantitative flow ratio (QFR) is one of those techniques, that overcomes the abovementioned limitations. QFR is a novel vascular assessing modality, that allows rapid measurement of FFR, based on a 3-dimensional angiographic reconstruction and the flow dynamics algorithms-contrast flow frame count. 3-dimensional quantitative angiography is obtained by selecting 2 diagnostic angiography projections, at least 25° apart [25]. Most importantly, the whole procedure is performed with neither wire nor hyperemia. Therefore, it indirectly answers the question of what the physiological severity of epicardial stenosis is, due to evaluating the morphology of the examined artery. Clinical trials show significant correlation and agreement between QFR and FFR measurements of the post-PCI patient. Both techniques may be effective in the assessment of suboptimal coronary stenting identification [25,26,27,28,29]. Additionally, large prospective studies are ongoing with a specific focus on clinical follow-up and prespecified angiography acquisition protocols [30]. Computing software innovations allow for an increasingly accurate evaluation of non-invasive FFR. Techniques such as vessel fractional flow reserve (vFFR) and angiography-derived fractional flow reserve (FFRangio) correlate well with the FFR values obtained by conventional means and are characterized by a high diagnostic accuracy to detect FFR ≤ 0.80 (Figure 3) [31,32,33]. 

The DOCTOR study compared QFR before PCI to post-stenting FFR and post-stenting QFR to post-PCI FFR in patients with NSTEMI-ACS. The analysis had a good correlation. Biscaglia et al. proved that post-PCI QFR lower than 0.90 was related to a higher risk of cardiovascular death, myocardial infarction and ischemia-driven revascularization connected with target vessel [34]. IVUS-guided treatments were associated with less frequent 2-year vessel-oriented composite endpoints (VOCEs) [35]. When comparing patients with normal and reduced left ventricle ejection fraction (LVEF), those with reduced seem to have more benefits after successful revascularization. A greater increase in post-PCI QFR value and LVEF was observed [36]. In the PANDA III study, researchers screened patients with diabetes mellitus (DM). The achievements of post-PCI were comparable in both DM and non-DM. Vessels with low post-PCI QFR (<0.92) were proportionately connected with an increased risk of 2-year VOCE [37].

Computed tomography-derived flow fractional reserve (FFR-CT) is also based on imaging techniques to derive FFR. Recent improvements in computational fluid dynamics (CFD) and mathematical models have made it possible to obtain measurements of coronary flow and pressure from computed tomography angiography (CTA) of coronary vessels. In addition, CTA allows assessment of the forces exerted on the vessel wall called wall shear stress (WSS) [38,39]. Three principles form a basis for this FFR computing. The first one claims that baseline coronary flow depends on the myocardial oxygen demand. According to the second one—the resistance of the microcirculatory bed at rest is inversely, not linearly, proportional to the size of the feeding vessel. Finally, the third one defines, that the coronary microcirculation has a predictable response to adenosine, which is produced from the breakdown of ATP when the oxygen supply to myocardial cells is reduced [40,41]. Investigators have shown that FFR-CT is strongly correlated with invasively pressure-wire-derived FFR [42,43,44]. PACIFIC study also reported an improved diagnostic discriminative ability compared with coronary CTA, single-photon emission computed tomography (SPECT), and positron emission tomography (PET) [45]. 

## 4. Optical Coherent Tomography

Optical coherent tomography (OCT) is an invasive imaging modality, which provides rapid acquisition of coronary artery cross-sectional images. Similarly, to IVUS, OCT also necessitates a catheter advanced into the coronary artery. However, these two visualization systems differ significantly—OCT uses a light source in the near-infrared spectrum instead of ultrasounds. It implicates the properties of the resulting image and the possibility of visualizing changes in the vessels. While the axial resolution of IVUS ranges between 150 and 200 microns, OCT enables acquisition in a ten times higher range, i.e., 12–18 microns [46]. This allows for a finer visualization of the lumen and the arterial wall layers—intima, media, and adventitia—where coronary pathology requiring evaluation is most prevalent [9]. Oppositely, near-infrared light penetration is limited to 1–3 mm compared with 4–8 mm achieved with IVUS, except for heavily calcified plaques, in which ultrasounds propagation is limited [46]. It is also worth highlighting the usefulness of this modality in evaluating and optimization of stenting after PCI. The high image quality and resolution allow for assessment of the conditions of stent placement wherein either coronary angiography or FFR examination is obstructed [29].

OCT, as high-resolution intravascular imaging for the rapid evaluation of stent coverage, apposition and detailed characterization of neointimal tissue and vessel wall pathology, can provide information about mechanical abnormalities and help guide the management of CAD [47,48]. OCT-guided PCI assesses plaque preparation, lesion length, segmental reference sizes, lesion coverage, stent expansion, malapposition, wire positions, and ostial results in order to provide adequate vessel and stent expansion, full stent apposition, and optimal lesion coverage [49]. The underexpansion of stented segments can provide the base of in-stent restenosis causing suboptimal results after PCI but may be solved by different techniques such as the proximal optimization technique or by modifying fibrocalcified plaques sufficiently before implanting stents. OCT enables checking for underexpanded stents and assessing the potential need for a proximal optimization technique [49,50]. OCT and IVUS guidance show a similar degree of stent expansion with a low frequency of major stent malapposition, tissue prolapse, and edge dissections [51]. Underexpansion is one of the leading causes of acute stent thrombosis (ST), a rare phenomenon, but associated with serious clinical consequences [52,53]. However, cases of ST have different pathophysiology with increasing time from PCI and have a variety of factors affecting this process [54,55]. From the clinical perspective understanding the underlying pathophysiological process leading to ST is crucial and OCT makes that goal feasible [55]. This intravascular technique detects an underlying morphological abnormality in 97% of cases of confirmed ST [48]. Recent researches show that one of the main components of pathophysiology of very late ST is neoatherosclerosis which is de novo process of atherosclerosis in the neointimal region of the stented segment [56]. OCT providing information about macrophage infiltration, lipid accumulation, in-stent calcification, or neointimal rupture, exert a key role in this regard assessment of neoatherosclerosis [48,56].

The mentioned above characteristics of OCT led investigators to use this technique to reconstruct the vessel geometry to reproduce FFR values. Owing to mathematical algorithms pressure loss across stenosis can be calculated by diagnostic software. The OCT-derived FFR, called optical flow ratio (OFR), provides the ability to assess physiological aspects of the obstruction with simultaneous morphology imaging in a single pull-back. OFR correlation with conventional wire-derived FFR was observed as excellent, by extending the OCT acquisition procedure by just a few minutes [6,57,58,59,60]. OFR demonstrated superiority over QFR in determining physiological the significance of coronary stenosis [61,62,63]. 

Among all characterized modalities above, OFR tends to be the most feasible technique regarding its compliance and predominance with alternative morphophysiological assessments. Recent reports support this method, suggesting it could facilitate everyday clinical practice [62]. 

However, it is worth mentioning that the role of coronary intravascular imaging is limited in everyday clinical practice. A survey among 1105 interventional cardiologists was conducted and the high cost of intravascular imaging was the most commonly reported reason limiting the clinical use of these visualization techniques [64]. Even though, unlike ultrasound, OCT catheters contain no transducers within their frame, they are more expensive than IVUS catheters [65,66,67]. The lack of coverage for such procedures by most insurance companies causes practical difficulties on the data acquisition front as well [68]. Nonetheless, the added value of this modality is constantly better recognized and in some countries is refundable by the insurance companies [69]. OCT has initial incremental increases in expenses but also is a more cost-effective diagnostic strategy than IVUS along with coronary angiography for patients with CAD [65]. 

## 5. OCT versus FFR in Clinical Trials

Since so far mostly conventional coronary angiography has been separately compared with either intracoronary imaging or physiologic vascular assessment and their impact on PCI outcomes [70,71]. Relatively few studies have compared OCT with FFR examination. Follow-up length, study design, and location of examined obstructive lesions varied among individual studies, and different therapeutical management were compared [72,73,74]. Due to the number of dedicated trials comparing OCT with FFR Burzotta et al. carried out The Fractional Flow Reserve vs. Optical Coherence Tomography Guide to Revascularization of Intermediate Coronary Stenoses (FORZA) trial [72].

The above-mentioned study sought to examine the rate of significant residual angina and major adverse cardiovascular events after performing on intermediate stenotic coronary artery either OCT- or FFR-guided PCI, depending on randomization. FFR guidance coronary procedure was indicated when the value of <0.80 was measured on the targeted angiographically intermediate coronary lesion. This group underwent PCI in order to achieve an FFR equal to or above 0.90. Patients with primary FFR higher than 0.80 were treated with optimal medical therapy only. Accordingly, in an OCT guidance there had to be at least one of the three criteria present. The following were: stenosis area  ≥ 75%, stenosis area from 50% to 75% with a minimal lumen area < 2.5 mm^2^, and major plaque ulceration evidence in OCT [72]. As OCT enables to visualize in high resolution, such stenting disturbances as underexpansion, malapposition, uneven stent strut distribution, or small intra-stent thrombotic formations can be observed. As a consequence, in the case of impaired stent implantation, OCT guidance also includes further optimization [74,75]. The purpose of the study was defined as a prospective assessment of improving symptoms, the recurrence of residual angina, and the rate of major adverse cardiovascular events, which included all-cause death, myocardial infarction, and target vessel revascularization, at thirteen months follow-up as the primary endpoint. The further secondary endpoint was the global cost evaluation of a particular strategy at one- and thirteen-month follow-up [72].

The investigators found at one-month follow-up, that in the group randomized to OCT guidance, a significantly lower number of patients was indicated for treatment with optical medical therapy and a higher number of stents per patient was used (*p* < 0.0001). Additionally, OCT was more frequently used after PCI than FFR to optimize stent implantation, but this finding was insignificant (*p* = 0.09), and consequently, an optimal stenting effect was more commonly achieved after OCT-guided PCI than FFR. Furthermore, OCT guidance was associated with higher iodine-based contrast consumption and as a result higher rate of contrast-induced acute kidney failure, not required in any of the cases of hemodialysis. According to the primary endpoint, researchers discovered that the rate of significant residual angina and major adverse cardiac events (MACE) was slightly different in favor of FFR but statistically insignificant (*p* = 0.84). Moreover, a trend toward longer hospitalization and higher procedural costs were associated with OCT guidance in comparison to FFR (0.07 and <0.0001, respectively) [76].

In general, initial observation showed a greater benefit of performing FFR-guided PCI in terms of economic issues and less frequent renal insufficiency resulting from decreased contrast supply during the procedure. The researchers concluded that despite more PCIs performed in the OCT group than in the FFR group, clinical outcomes were not significantly affected, at least in this follow-up period of one month. This study period could have supported better guidance of FFR, but the results of the study were to come after a second follow-up at 13 months [77].

The clinical outcomes at 13 months presented quite differently. MACEs and significant residual angina occurred more frequently in the FFR group in comparison to the OCT one. Procedural costs and the number of patients that continued to be managed with optimal medical therapy remained higher in the OCT group. Over 13 months in both groups, a steady improvement in angina status was reported [77]. 

The considered study presents long-term results of the clinical management depending on the chosen diagnostic modality. However, like any clinical trial, this is not without limitations. First of all, as earlier described, these two techniques provide answers to different questions. Therefore, they should not be juxtaposed with each other but considered complementary. What is more, the investigation took the form of an unblinded trial or otherwise known as open-label. From the definition, the consequence of this could lead to biases regarding mostly target vessel revascularization, which was included among the major adverse cardiac events of this study. Another matter is the rate of MACEs that occurred during follow-up. Those that occurred after a longer period of observation may seem less significant. When considering MACEs, we should pay attention to the dual antiplatelet therapy used after the percutaneous procedure. The more patients undergo PCI, the more receive therapy, which results in lower cardiovascular event prevalence in those in the OCT group [78,79]. 

## 6. Intravascular Ultrasound

The significant role of IVUS in contemporary stent-based PCI is invaluable. This imaging tool brings more detailed information about lumen size and vessel wall thickness, assesses plaque composition, selects proper stent sizes, and optimizes stent expansion, apposition, and geographic miss [80]. Doppler guidewire velocimetry provides clinicians with measurements of post-stenotic flow velocity continuously during the coronary intervention, previously unavailable from earlier studies using larger Doppler catheters [81]. IVUS systems for clinical use can be divided into 2 main types: the mechanical single-element rotating transducer and the solid-state electronic phased array transducer. The advantage of the solid-state catheter over rotational systems includes enhanced trackability and lack of non-uniform rotational distortion artifacts [80]. 

The current gold standard of lesion-specific coronary revascularization decisions in patients with stable CAD-FFR is well-correlated with values measured using established equations and accurate 3-dimensional IVUS imaging [43,82]. This invasive visualization technique became a new standard in cases with younger patients, who have less high-risk clinical features, and have more complex lesions such as left main and multivessel disease in which IVUS yields the largest benefit in reducing MACE and target-lesion revascularization [83,84]. 

In cases of left main coronary artery stenosis (LMCS), accurate lesion assessment is crucial in guiding clinical management [85]. Underestimation of the significance of LMCS beholds the risk of inappropriate deferral of revascularization in cases in which stenosis is associated with a poor long-term outcome, whereas overestimation of mild to moderate LMCS stenosis provides unnecessary interventions and may lead to the premature closure of either the native vessel or the graft [86,87]. Unfortunately assessing angiographic lesion severity localized in the left main coronary artery is associated with high interobserver variability and low agreement (41% to 59%) [88,89]. Difficulty in the assessment of LMCS-induced application of new visualization techniques such as IVUS which can evaluate stent under-expansion, incomplete lesion coverage, small stent area, large residual plaque, and stent malapposition, which have been found to predict stent thrombosis after stent placement [90,91]. IVUS application in the assessment of lesions of the left main coronary artery significantly reduced the risks of all-cause mortality by ~40% and cardiac death by 53% compared with conventional angiography-guided PCI [92]. This intravascular imaging technique cause usage of more appropriately sized stents and indicate a lower risk of subsequent stent thrombosis [93]. IVUS became an excellent diagnostic tool in cases of LMCS in situations such as LV dysfunction where maximal hyperemia may not be achieved [94]. IVUS guidance is recommended for patients undergoing LMCA intervention by The European Bifurcation Club (EBC). This intravascular imaging technique provides clinically significant information before PCI about the risk of the side branch, optimal stent length, optimal stent diameters, and measurements of the proximal optimization technique [95]. Furthermore, this visualization technique can be used after PCI of LMCA in the assessment of residual edge stenosis, edge dissection, stent expansion and apposition, accidental abluminal rewiring, and other complications [95,96]. 

IVUS plays an important role in the recanalization of chronic total occlusion (CTO) during PCI, which remains a great challenge for clinicians [97]. This coronary artery visualization technique was associated with less in-stent late lumen loss and in-stent restenosis and ST when stenting occurred within the true lumen [98]. The risk of the composite of cardiac death or myocardial infarction and major adverse cardiac event rates were significantly lower in the IVUS-guided group than in the angiography-guided group during CTO-PCI [99].

IVUS-derived minimal lumen dimension (MLA) is a well-known strong predictor of MACE [87]. Despite that, the optimal cutoff value of an MLA remains debatable, and studies suggest values from 5.9 to 9.6 mm^2^ as the cut-off points of significant LMCS [100,101,102,103]. Most clinicians suggest that 6 mm^2^ is a safe value for deferring revascularization of the LMCA [103]. Apart from MLA, IVUS measurement of minimal lumen diameter (MLD) also became an important quantitative predictor of cardiac events [87]. Both MLA with the cut-off of 5.9 mm^2^ and MLD with the cut-off of 2.8 mm are extremely useful tools in the assessment of LMCS (sensitivity of 93%, specificity of 95% and sensitivity of 93%, specificity of 98%, respectively) [100]. Current ESC guidelines recommend IVUS for the risk stratification of patients with intermediate LM stenosis and assessment of cardiac allograft vasculopathy and plaque stability [13]. This visualization technique should be also considered in the detection of stent-related mechanical problems leading to restenosis [11]. 

IVUS-derived MLA accuracy is highly variable according to the localization of the lesion and shows a better correlation with FFR in LM lesions than in non-LM lesions [104,105]. Vessel size is the main factor that should be taken into consideration during the assessment of functional ischemia using MLA. The most accurate value for FFR < 0.8 correspond to MLA < 2.4 mm^2^ for lesions with reference vessel diameters of 2.5–3 mm, MLA < 2.7 mm^2^ in lesions with reference vessel diameters of 3–3.5 mm, and MLA < 3.6 mm^2^ with reference vessel diameters of >3.5 mm [11,106,107]. In addition to MLA’s moderate correlation to FFR values, MLD cut-off values in non-LM lesions are also problematic. Researches show that optimal sensitivity and specificity correspond to MLD < 1.8 mm in identifying significant lesions [108].

**Figure 4:**
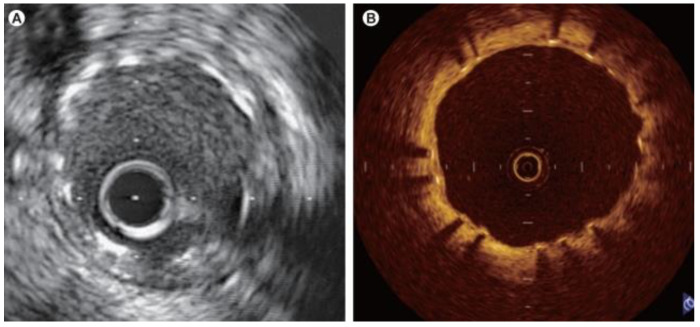
Images obtained at 7 months after implantation of a drug-eluting stent (Cypher; 3.0 mm × 18 mm) implantation: (**A**) IVUS, intravascular ultrasonic; (**B**) OCT, optical coherence tomography. Reproduced under a Creative Commons license from [115].

However, only a few data from large-scale multicenter studies for patients with CAD are available for current techniques of intravascular imaging such as IVUS or OCT, and procedural-based outcomes related to intravascular modality guidance compared with angiography guidance still require further investigation (Figure 4) [83,109,110,111,112,113,114,115].

## 7. Biomechanics and Plaque Stress

**Figure 5:**
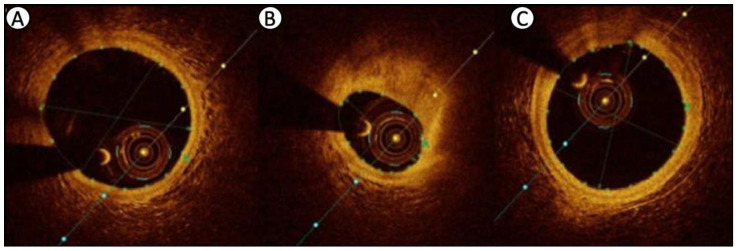
Optical coherence tomography cross-sectional images with measured lumen dimensions at various locations: (**A**) distal reference segment; (**B**) minimum lumen area; (**C**) proximal reference segment. Reproduced and adapted under a Creative Commons license from [128].

Rupture of an atherosclerotic plaque is the most frequent underlying cause of acute coronary syndromes (ACS), nevertheless, the mechanism of plaque rupture is not fully understood [116]. A variety of components participates in setting the stage for rupture, calling attention to the plaque composition and biomechanical factors [116,117]. Studies have identified several structural characteristics of rupture-prone plaques: a large lipid pool inside a plaque, a thin fibrous cap (<65 μm), and increased macrophage infiltration within the cap [117,118,119]. Biomechanical factors that may predispose a plaque to rupture are hemodynamic shear stresses, turbulent pressure fluctuations, transient compression, mechanical shear stresses, sudden increases in intraluminal pressure, rupture of the vasa vasorum, and tensile stress concentration within the wall of the lesion [116,120,121,122,123,124,125]. Both biomechanical and structural elements require high-resolution imaging techniques [126]. OCT as a new imaging method with a resolution of 10–20 μm is correlated well with the histological examination and easily demonstrates the internal elastic laminae, which cannot be identified by IVUS (Figure 5) [126,127,128]. In addition, OCT shows the superior delineation of vessel layers and ring-down artifacts do not occur compared with IVUS [129]. OCT images can be also used for assessing plaque structural stress (PSS) over the cardiac cycle. That technique embraces automatic plaque characterization and finite element analysis (FEA) by a fully automated process connected with artificial intelligence [130]. FEA is an engineering technique that divides complex structures into smaller segments (finite elements) and using powerful computers, allows for the calculation of the distribution of stress within the original complex structure [116,131]. 

Permanently pulsating pressure waves in cardiac vessels generate strain and influence plaque caps resulting in fatigue and breakage [116]. Thereupon, assessment of cyclical plaque structural stress may increase the prognostic value for cardiac events to prevent forthcoming ACS. As it happens, even fine-scale changes such as lumen diameter or thickness of fibrous cap induce significant variation in PSS [130]. Furthermore, the cap and necrotic core thickness have the most significant influence on the calculation of stresses [132]. Likewise, the vascular structure defined by OCT presents a relevant basis for biomechanical analysis [133]. Studies have shown that high plaque stress correlates with plaque rupture and acute coronary syndromes [128,134]. In normal coronary segments, substantially lower ΔPSS occurs, compared with normal and diseased coronary segments in the examination. By the same token strain and plaque stress in the proximal segments are larger than in the distal segments of the lesion and that equals more often plaque ruptures in proximal segments. Moreover, ΔPSS positively correlates with lipidic plaque area (LPA) and negatively with fibrous cap thickness, in such a way corroborating a direct connection between ΔPSS and morphologically rupture-prone plaque. That affirms the importance of plaque morphology and composition in establishing the actual plaque stress [130]. Assessment of coronary lesions using OCT to compute proximal shoulder and MLA can lead to optimal therapeutic management decisions reducing the risk of future adverse events [135].

Considering the application of PSS to assess morphology and risk of complications of ruptured atherosclerotic lesions, this parameter should be used as a complementary tool in the diagnostic process. By supplementing imaging studies, such as IVUS, with PSS, we can estimate with greater accuracy the incidence of MACE resulting from ruptured lesions and, especially, from non-culprit lesions, as they are more frequent in coronary vessels [134,136]. However, the relationships between lesion sites, properties, and sizes of plaques and vessels are complex and may affect PSS values in different ways [136]. The results are encouraging, but further studies are required to realize the true potential of PSS in the diagnostics of coronary atherosclerosis.

Interest in CFD increased with the wider availability of FFR-CT, which allowed no-invasive measurement of WSS, in contrast to previous modalities that remained invasive techniques such as IVUS and angiography [137]. The WSS is a force on the vessel wall and its oscillation mainly affects the line of first contact between the blood and the vessel tissue, which are the endothelial cells. The cell phenotype shows a tendency to convert towards proatherogenic cells through the higher accumulation of low-density lipoprotein cholesterol, oxidative stress, and activation of inflammatory reaction and consequent atherosclerotic progression [138]. Restenotic and early atherosclerotic lesions are identified with low and oscillatory WWS, leading mainly to lesions at sites such as bifurcations, ostia, or vessel suture sites in the case of CABG. In contrast, late atherosclerosis and endothelial erosion are a consequence of both high and low values. Further, plaque rupture is localized to areas with increased values [139,140].

Studies have shown a correlation between WWS and plaque progression, properties, and distant prognosis of complications [139,141]. For low WWS, an association with future revascularization of the vessel stented with bare metal stent and MACEs related to non-culprit lesions has been demonstrated [134,142]. High WWS values have been associated with increased plaque vulnerability, thrombogenic potential, and the eventual occurrence of plaque-related events such as myocardial infarction [143]. In addition, the studies show the incremental prognostic value over FFR of WSS measured in the proximal segments of lesions to predict myocardial infarction in patients with stable CAD [144]. According to experts’ opinions, in clinical practice, the best modalities to assess the impact of WWS on plaque both characteristics and progression, and stented areas remain measurements based on CTA or OCT/IVUS fusion with biplane angiography. The equal placement of invasive and non-invasive techniques should be carefully considered in the diagnostic process, keeping in mind the possible complications of invasive modalities. In low-risk patients, CTA is recommended over invasive methods, which have limited use in this group [139]. A broader application of WWS as an adjunctive modality may assist diagnostic management by allowing more accurate detection of at-risk patients and estimation of the natural history of atherosclerotic plaque. However, in clinical practice, we should remember the primacy of widely approved diagnostic tests.

## 8. Conclusions

Optimal assessment of coronary stenoses continues to represent a significant clinical challenge. The development of invasive and non-invasive visualization techniques provides useful hybrid tools which after proper validation can guide the optimal management of intermediate coronary lesions. For clinicians, it is crucial to have a thorough understanding of these imaging tools which are expected to be increasingly advocated in the upcoming guidelines and recommendations.

## Figures and Tables

**Table 1:** Currently available non-hyperemic pressure ratios (NHPRs).

NHPR	Calculation	Period of the Cardiac Cycle	Threshold
Instantaneous wave-free ratio (iFR)	Pd/Pa calculated during the WFP ^2^ within diastole.	WFP ^2^ in diastole	≤0.89
Resting full-cycle ratio (RFR)	The lowest Pd/Pa ^1^ over the entire cardiac cycle. Mean of 4–5 consecutive cycles.	Whole cycle	≤0.89
Diastolic hyperemia-free ratio (DFR)	Average Pd/Pa ^1^ over the approximated diastolic period averaged over five consecutive cardiac cycles.	Diastole	≤0.89
Resting Pd/Pa ^1^	Resting Pd/Pa ^1^ averaged over the entire cardiac cycle.	Whole cycle	≤0.91

^1^ Pd/Pa, ratio of mean distal coronary artery pressure to mean aortic pressure in the resting state; ^2^ WFP, wave-free period. Reproduced and adapted under a Creative Commons license from [19].

## Data Availability

Not applicable.
